# Strain-Dependent Thermoadaptation in the Fish Pathogen *Aeromonas salmonicida* subsp. *salmonicida*

**DOI:** 10.3390/microorganisms13092171

**Published:** 2025-09-17

**Authors:** Kim C. Fournier, Pierre-Étienne Marcoux, Antony T. Vincent, Steve J. Charette

**Affiliations:** 1 Institut de Biologie Intégrative et des Systèmes, Université Laval, Quebec City, QC G1V 0A6, Canada; kim.fournier.3@ulaval.ca (K.C.F.); pierre-etienne.marcoux.1@ulaval.ca (P.-É.M.); antony.vincent@fsaa.ulaval.ca (A.T.V.); 2 Département de Biochimie, de Microbiologie et de Bio-Informatique, Faculté des Sciences et de Génie, Université Laval, Quebec City, QC G1V 0A6, Canada; 3 Département des Sciences Animales, Faculté des Sciences de l’Agriculture et de l’Alimentation, Université Laval, Quebec City, QC G1V 0A6, Canada

**Keywords:** *Aeromonas salmonicida* subsp. *salmonicida*, thermoadaptation, A-layer, *AsaGEI*, type III secretion system, lifestyle

## Abstract

Strains of *Aeromonas salmonicida* subsp. *salmonicida*, a major pathogen of salmonids, typically do not grow at temperatures above 30 °C. The effects of thermal stress on this bacterium have been extensively studied. Recently, we demonstrated that repeated exposure to cyclical thermal stress, reaching up to 37 °C, can induce permanent thermoadaptation in certain strains of this bacterium. Many aspects of this adaptation process remain poorly understood. We generated 88 thermoadapted strains of *A. salmonicida* subsp. *salmonicida* capable of sustained growth at 33 °C or higher demonstrating that prolonged heat exposure can shift a substantial proportion of psychrophilic strains toward a more mesophilic-like behavior. Although growth at 35 °C was still weaker than in naturally mesophilic *A. salmonicida* strains, some thermoadapted strains were able to grow up to 37 °C. North American strains harboring the genomic island *AsaGEI1a*, a known biomarker, exhibited a significantly reduced capacity for thermoadaptation, suggesting a possible genetic constraint, although genomic analyses indicate that *AsaGEI1a* itself is not directly responsible for this limitation. Genotyping and phenotypic analyses revealed that thermoadaptation is frequently associated with the loss of Type III secretion system and the A-layer, two key virulence factors. Only 7% of the thermoadapted strains retained both features. Overall, our findings suggest that thermoadaptation may represent a potential mechanism influencing the persistence of some psychrophilic *A. salmonicida* subsp. *salmonicida* strains in warming aquatic environments under climate change.

## 1. Introduction

The *Aeromonas* genus includes bacteria with diverse lifestyles, classified into two main categories based on growth temperature: mesophilic strains (*A. hydrophila*, *A. veronii*, etc.) capable of growing at 37 °C, and psychrophilic strains, as *A. salmonicida* subsp. *salmonicida*, which cannot tolerate this temperature [1,2]. This last bacterium is the causative agent of furunculosis in cold-water salmonids [1].

*A. salmonicida* subsp. *salmonicida* produces a brown diffusible melanin pigment at 22 °C and below [3,4] and relies on multiple virulence factors to establish infections, including the Type III secretion system (TTSS) and the A-layer. The TTSS, encoded mainly on the pAsa5 plasmid, is a needle-like structure that injects effector proteins into host cells, disrupting their cellular functions [5,6]. Strains lacking the TTSS cannot cause infection [7,8].

The A-layer is a tetragonal protein network, anchored to the cell by lipopolysaccharides (LPS), which covers the bacterial surface. It enhances virulence by promoting adhesion, auto-aggregation, and hydrophobicity while protecting against immune defenses and phages [9,10,11,12,13]. Its formation depends on the VapA protein (encoded by the *vapA* gene), which must reach the membrane surface and interact with the LPS. This process requires the AbcA transporter (encoded by the *abcA* gene), responsible for LPS synthesis and transport [14]. A decrease in bacterial virulence is observed when strains lack the A-layer [13].

The genome of *A. salmonicida* subsp. *salmonicida* harbors numerous mobile genetic elements (plasmids, prophages, insertion sequences, and transposons) that enhance genomic plasticity and virulence by promoting, among others, plasmid rearrangements, antibiotic resistance gene exchange, and the inactivation of certain virulence factors [5,15,16,17]. A specific group of genomic islands, of approximately 50 kb in size and named *AsaGEI*, has also been identified in this bacterium. The role of *AsaGEIs* remains unknown. They predominantly encode phage-related proteins. So far, six types of *AsaGEIs* have been described (*1a*, *1b*, *2a*, *2b*, *2c*, and *2d*), correlating with specific geographic distribution. *AsaGEI1a* and *2a* are found only in strains from North America. *AsaGEI2a* is also almost always associated with a prophage named Prophage 3 [18,19,20,21].

*A. salmonicida* subsp. *salmonicida* thrives below 20 °C and experiences genomic instability when exposed to temperatures above 22 °C. This heat stress promotes genetic instability, including rearrangements, particularly in the pAsa5 plasmid, leading to TTSS loss and reduced virulence [8,22,23]. By creating so-called rearranged strains, a previous study by our team showed that certain strains of this bacterium are more prone to TTSS loss. After prolonged exposure to 25 °C, Canadian strains were less likely to lose their TTSS than European strains, and TTSS excision was not induced in Canadian strains lacking *AsaGEI* or carrying *AsaGEI2a* and Prophage 3, likely due to the absence of a specific chromosomal gene cluster present in strains susceptible to TTSS locus excision. Among Canadian strains, those harboring *AsaGEI1a* were most prone to genomic rearrangements under thermal stress [24]. Beyond TTSS loss, temperature-induced genomic changes can also result in the loss of the A-layer, further diminishing virulence. These modifications may arise from insertion sequence transposition in *vapA* or *abcA*, as well as mutations or large-scale chromosomal rearrangements [9,25].

The isolation of mesophilic *Aeromonas salmonicida* strains has led to a reevaluation of the species’ taxonomy, emphasizing its genetic and ecological diversity [26,27,28,29,30]. These strains can grow efficiently from 7 °C to 37 °C, and even higher [26,29], whereas psychrophilic strains isolated from cold fish infection typically cannot survive at 30 °C and exhibit reduced growth at 7 °C and 18 °C compared to mesophilic strains. Phylogenetic analyses suggest that psychrophilic strains evolved from mesophilic ancestors, losing their ability to grow in mesophilic environments and adapting to a psychrophilic lifestyle [26,29,30,31].

Comparative genomics has already provided important insights into this evolutionary transition. Vincent et al. (2016) demonstrated that insertion sequences constrained psychrophilic strains to their cold-adapted lifestyle, limiting their genomic plasticity [26]. More recently, Long et al. (2023) identified genomic traits distinguishing psychrophilic and mesophilic lineages, such as lateral flagella, A-layer and T2SS proteins in addition to insertion sequences in psychrophilic strains, versus complete type IV pili in mesophilic strains [31]. Together, these studies highlight the genetic bases of temperature adaptation and confirm that psychrophilic and mesophilic strains are already well defined at the genomic level.

We recently demonstrated that two strains of *A. salmonicida* subsp. *salmonicida* can thermoadapt, i.e., gain the ability to grow at temperatures exceeding their initial upper thermal limit. The permanent thermoadaptation of *A. salmonicida* subsp. *salmonicida* strains was provoked by exposure to short thermal stress cycles reaching 37 °C [32]. Since *A. salmonicida* subsp. *salmonicida* is not commonly used as a biological model, virtually no data exist on its thermoadaptation. Understanding the thermal adaptation capacity of this bacterium is relevant, as it not only provides fundamental insights into the evolutionary transition from psychrophilic to mesophilic lifestyles, but also has important implications for the persistence, pathogenicity, and aquaculture impact of this pathogen in a changing climate.

This study aimed to address this gap by using the established thermoadaptation protocol and generating a collection of newly thermoadapted strains. These strains were characterized at both the phenotypic and genotypic levels, revealing a strain-dependent capacity to thermoadapt.

## 2. Materials and Methods

### 2.1. Bacterial Growth Conditions

The characteristics of the *A. salmonicida* subsp. *salmonicida* strains selected for this study are presented in Table S1. For each test performed, the different bacterial strains were thawed on tryptic soy agar (TSA) (Wisent, Saint-Jean-Baptiste, QC, Canada) plates and incubated at 18 °C for 72 h for psychrophilic strains and at 37 °C for 48 h for mesophilic strains. TSA was used as the solid growth medium, while tryptic soy broth (TSB) (BD, Mississauga, ON, Canada) was used for bacterial growth in liquid culture. For liquid culture experiments, precultures were prepared 24 h prior to the experiment by inoculating 10 mL of TSB with a single bacterial colony. The tubes were then incubated at 18 °C for 24 h with shaking at 200 RPM.

### 2.2. Production and Validation of Thermoadapted Strains

**Figure 1:**
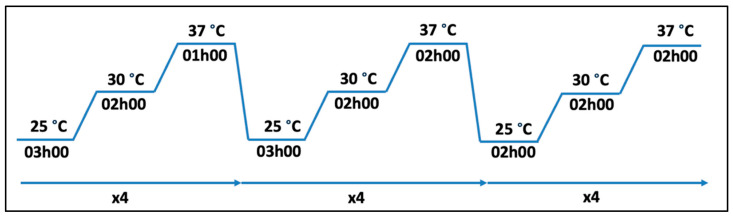
Temperature cycle used for the creation of thermoadapted strains.

The protocol used is based on previous work [32]. The parental bacteria (Table S1) were thawed, and precultures were prepared and incubated for 24 h at 18 °C with shaking at 200 RPM. On the day of the experiment, we measured the optical density of the liquid cultures with a spectrophotometer at 600 nm. The cultures were diluted to obtain an optical density of 0.1 in a final volume of 300 μL of TSB, then placed in a 48-well plate. We placed the plate in a Biotek Epoch2 microplate reader (Agilent, Santa Clara, CA, USA) and subjected the strains to temperature variations (25 °C, 30 °C, 37 °C) for 76 h (Figure 1).

**Figure 2:**
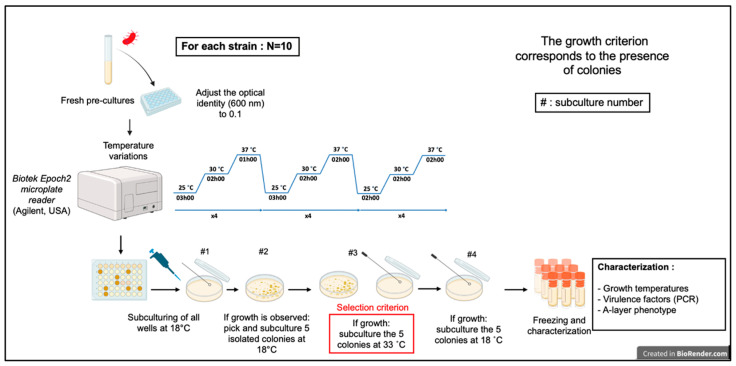
Schematic overview of the optimized thermoadaptation protocol.

After the thermoadaptation procedure, we cultivated four subcultures on TSA medium at various temperatures to confirm whether the selected strains had thermoadapted (Figure 2). The intention of the first subculture at 18 °C for 72 h was to verify bacterial survival after the thermoadaptation procedure. (step #2, Figure 2) For strains showing bacterial growth and pigmentation after this first incubation, five isolated colonies were subcultured a second time at 18 °C (72 h). The threshold of five colonies was set to reduce the workload and avoid analyzing multiple identical clones of the same bacterial strain. (step #3, Figure 2) The aim of the third subculture was to verify the thermoadaptation of the bacteria. Since most of the selected parental strains could grow up to 27 °C (Table S1), the selection criterion for thermoadaptation was set at 33 °C, which is 6 °C above the maximum temperature that the most parental strains grew at. The third subculture was incubated for 72 h, and we checked the growth every 24 h. (step #4, Figure 2) Finally, the last subculture at 18 °C (72 h) aimed to obtain a sufficient bacterial biomass for freezing thermoadapted strain stocks. In the end, colonies capable of growing at 33 °C or higher were selected for freezing (Figure 2). All thermoadapted strains produced in this study were inoculated into 1 mL of LB medium (Wisent, St-Bruno, QC, Canada) containing 15% glycerol (in a freezing tube) to prepare the bacterial stocks. These were then frozen at –80 °C until needed.

The growth capacity of the thermoadapted strains and their parental strains was tested on a solid medium. For the thermoadapted strains, three temperatures were tested (30, 35, and 37 °C), in addition to those verified during the protocol for producing these strains (18 °C and 33 °C). For each temperature, the bacteria were thawed on a TSA medium and incubated at the tested temperature.

### 2.3. Bioinformatics Analyses

#### 2.3.1. Comparison of *AsaGEI1a* Sequences

We sequenced strains M13566-12 and M17930-12 using MiSeq technology (Illumina, San Diego, CA, USA) at the Plateforme d’Analyse génomique of the Institut de Biologie Intégrative et des Systèmes (Université Laval, Quebec City, QC, Canada). The sequencing reads were filtered using FastP (version 0.23.4), and assemblies were performed with Shovill (version 1.1.0) [33,34]. The contigs were aligned to the reference genome of the strain A449 (GCA_000196395.1) using the CONTIGuator tool (version 2.7.5) [35]. The *AsaGEI1a* sequence of strain 01-B526, available on NCBI (GCA_003692675.1), was then used as a reference to locate the position of *AsaGEI1a* in the M13566-12 and M17930-12 strains, using Blastn 2.16.0 [36]. The *AsaGEI1a* sequences from strains M13566-12 and M17930-12 were extracted and compared using Blast [36]. These sequences were also compared to the *AsaGEI1a* from the reference strain 01-B526. The sequence of *AsaGEI1a* from strains M13566-12 has been deposited on Genbank (accession number PV737463).

After growth on TSA at 18 °C for 72 h, the DNA from strain M17930-12 was extracted from colonies using DNeasy Blood and Tissue Kit (Qiagen, Toronto, ON, Canada) according to the manufacturer’s protocol. Bacterial genome sequencing was performed by Plasmidsaurus (https://www.plasmidsaurus.com/) using Oxford Nanopore Technology (ONT). Assemblies were de novo generated by Plasmidsaurus as described on their website. The chromosomal sequence of strain M17930-12 has been deposited on Genbank (accession number PRJNA1269977).

#### 2.3.2. Genome Comparison from Different Groups

To determine if other genomic elements (besides *AsaGEIs*) might be involved in the thermoadaptation of the bacterium, the genome sequences of strains possessing an *AsaGEI1a* were compared to those of strains with an *AsaGEI2a* and those without *AsaGEIs*, with the aim of identifying genes unique to each strain group. In total, we analyzed 15 strains (Table S2). The sequences were annotated with Prokka (version 1.14.6) [37], and a Python script using Diamond (version 2.1.9) was used to align the protein sequences and keep the best alignment sequence with a maximum e-value of 10-10. Then, the script identifies proteins specific to each strain group [38]. The identified proteins were then individually compared to sequences from the nr/nt database of the National Center for Biotechnology Information (NCBI) using BLASTp [36]. This analysis was also performed with strain M13566-12 genome compared to the genome groups described above using the same strategy.

### 2.4. PCR Amplification and Electrophoresis

DNA lysates for the strains analyzed were obtained using a previously described protocol [15]. PCR amplifications were carried out on thermoadapted strains to verify the presence of genes that code for certain virulence factors in the bacteria, namely the *abcA* and *vapA* genes for the A-layer and the *ati2* gene for the TTSS locus. The reaction mix was prepared according to the manufacturer’s recommendations for a 20 μL PCR reaction as previously described [19]. The primers used for PCR amplification are listed in Table S3. PCR conditions were as follows: for genes associated with the A-layer, the reactions were initiated with a denaturation step at 95 °C for 2 min 30 s, followed by 30 cycles of 30 s at 95 °C, 30 s at 55 °C, and 1 min at 68 °C. A final extension was performed at 68 °C for 5 min. For genes related to the TTSS, the reactions were initiated with a denaturation step at 95 °C for 2 min 30 s, followed by 30 cycles of 30 s at 95 °C, 30 s at 55 °C, and 30 s at 68 °C, with a final extension of 5 min at 68 °C. PCR products were verified by agarose gel electrophoresis using a 1% agarose gel containing 0.5 μg/mL ethidium bromide. Gels were visualized under ultraviolet light. All PCR reactions were performed in duplicate.

### 2.5. Detection of the A-Layer

To assess the presence of the A-layer in thermoadapted strains, a phenotypic assay using a liquid Coomassie blue medium was performed following an established protocol to detect VapA proteins, which constitute the A-layer on the bacterial surface [39].

## 3. Results and Discussion

In our preliminary work, we investigated thermotolerance in *A. salmonicida* subsp. *salmonicida* by gradually increasing incubation temperatures on solid media. This method did not lead to stable, heritable adaptation. However, an alternative approach involving thermal cycling with temperatures up to 37 °C (Figure 1 and Figure 2) in liquid media successfully produced some permanently thermoadapted strains. This method was originally developed to use elevated temperature as a selection marker in conjugation experiments [32].

**Figure 3:**
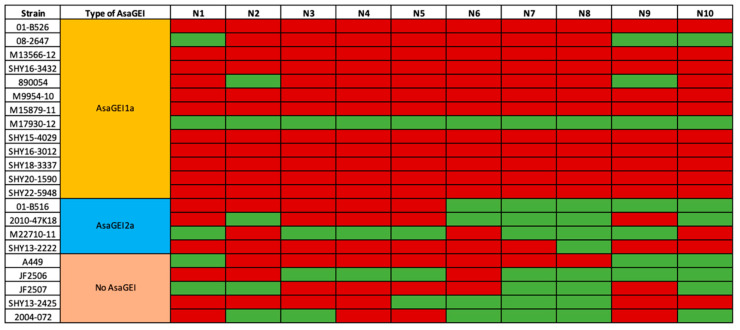
Thermoadaptation trial results for parental strains, grouped according to their *AsaGEI* type. Green indicates successful thermoadaptation, while red indicates failure. Thermoadaptation was considered unsuccessful if no colony growth occurred after Subculture #1 (see Figure 2). The first five strains carrying *AsaGEI1a* were tested in an initial round, while the eight additional strains from the same group were evaluated in a subsequent round.

Beyond its technical utility, the thermoadaptation process itself is of considerable biological interest, particularly in the context of the psychrophilic/mesophilic dichotomy observed within the *A. salmonicida* species. To gain a deeper understanding of this phenomenon, we undertook a systematic investigation to evaluate the thermoadaptive capacity of multiple *A. salmonicida* subsp. *salmonicida* strains. Specifically, we examined the relationship between thermoadaptation and the presence of *AsaGEIs*, as we had previously done when analyzing pAsa5 plasmid rearrangements and the loss of the TTSS during growth at 25 °C [24]. Thermoadaptation assays were conducted using five strains harboring *AsaGEI1a*, four strains carrying *AsaGEI2a*, and five strains lacking any *AsaGEI* element (Figure 3). To ensure the detection of consistent trends between groups, ten biological replicates were performed for each strain. A clear pattern emerged from the trials, revealing a reduced capacity for thermoadaptation in strains carrying *AsaGEI1a* (Figure 3). To confirm this trend, eight additional strains carrying *AsaGEI1a* were tested using the same thermoadaptation protocol. Of the 130 trials involving *AsaGEI1a*-positive strains, only 15 (11.5%) successfully yielded thermoadapted strains. Notably, 10 of these successful outcomes originated from a single parental strain, M17930-12. In contrast, higher thermoadaptation success rates were observed in strains carrying *AsaGEI2a* (45%, 18/40) and in those lacking any *AsaGEI* element (50%, 25/50). For these two groups, all strains tested gave thermoadapted strains in at least three trials except for strain SHY13-2222 which possesses an *AsaGEI2a* and thermoadapted in only one trial.

Logistic regression analysis supported these observations. Using *AsaGEI1a* as the reference, strains carrying *AsaGEI2a* displayed ~6-fold higher odds of successful thermoadaptation (odds ratio, OR = 6.3; 95% confidence interval, 95% CI: 1.1–35.4; *p* = 0.038, where the OR measures the relative likelihood of adaptation compared to the reference, the 95% CI indicates the range of plausible values for the OR, and the *p*-value estimates the probability that the observed difference is due to chance), and *AsaGEI*-negative strains showed ~8-fold higher odds (OR = 7.7; 95% CI: 1.6–36.9; *p* = 0.011). When excluding strain M17930-12 from the analysis due to its atypical thermoadaptation profile, the estimated success rate for *AsaGEI1a*-positive strains decreased further to 4.2%, and the statistical contrasts became even stronger, with *AsaGEI2a* strains showing ~19-fold higher odds (OR = 18.8; 95% CI: 3.6–98.1; *p* < 0.001) and *AsaGEI*-negative strains ~23-fold higher odds (OR = 23.0; 95% CI: 5.2–101.3; *p* < 0.001). Together, these results demonstrate that the presence of *AsaGEI1a* is strongly associated with a reduced capacity for thermoadaptation.

To determine whether *AsaGEI1a* itself was responsible for limiting thermoadaptation, we compared its sequence from strain M17930-12, which successfully thermoadapted in all assays, with those from two non-thermoadapting strains, M13566-12 and 01-B526 (Figure 3). These analyses revealed several dozen nucleotide differences between the *AsaGEI1a* sequences of strains M13566-12 and 01-B526. Notably, the *AsaGEI1a* sequence in strain M17930-12 was 100% identical to that of strain M13566-12, which consistently failed to thermoadapt. This finding suggests that *AsaGEI1a* alone does not directly impair thermoadaptation.

We therefore explored whether other genomic elements in strains carrying *AsaGEI1a* might explain the observed association. To investigate this, we compared the genomes of three groups of strains: those carrying *AsaGEI1a*, those with *AsaGEI2a*, and those lacking any *AsaGEI* (Table S2), with the aim of identifying unique genes that could account for the reduced thermoadaptive capacity linked to strains that bear *AsaGEI1a*. The analysis revealed that *AsaGEI1a*-carrying strains possess two unique genes not present in the other two groups (Table 1). These genes encode distinct proteins and are located at different genomic positions, none of which are in proximity to the *AsaGEI1a* insertion site. Strains that bear *AsaGEI2a*, despite their capacity to thermoadapt, only share one unique gene compared to the two other groups, which encodes a tyrosine recombinase (XerC). This gene is frequently found in the genome. Similarly, strains lacking any *AsaGEI* do not differ from those with *AsaGEI2a* in terms of unique gene content, although they do possess four genes absent from strains carrying *AsaGEI1a*, three of which encode hypothetical proteins. Based on the predicted functions of these genes, the genomic analysis does not provide clear insight into their potential involvement in thermoadaptation. When compared to the genome sequences of other *AsaGEI1a*-positive strains, the genome of strain M13566-12, which displayed thermoadaptation, did not contain any unique genes.

It also remains possible that point mutations scattered throughout the genome, which may affect the activity of certain genes, contribute to the thermoadaptation phenotypes observed. For example, Hug and Gaut (2015) identified many point mutations, scattered throughout the *Escherichia coli* genome associated, with thermoadaptive phenotypes following experimental evolution at 42.2 °C [40]. To gain deeper insights into this aspect, a more comprehensive analysis involving a large number of genomes from both thermoadapting and non-thermoadapting strains of *A. salmonicida* subps. *salmonicida* will be required. Distinguishing phenotype-relevant mutations from those with no functional impact is inherently challenging. Furthermore, it is likely that multiple different genes, such as those involved in the same metabolic or signaling pathway, may mutate in functionally non-equivalent ways across strains, yet still result in similar phenotypes. This complexity underscores the need to analyze hundreds of genomes to identify meaningful patterns and potential determinants of thermoadaptation, including insertions and deletions (indels) as well as single nucleotide polymorphisms (SNPs) particularly in stress-response pathways.

Thermoadaptation trials revealed that some bacterial strains were unable to adapt to higher temperatures under the standard 76 h procedure used in this study, as subculturing following the cycle showed no viable bacteria (Figure 1). We tested whether a shorter cycle, half the original duration, could promote thermoadaptation (Figure S1). Two strains that had never successfully thermoadapted (01-B526 and SHY16-3432) were tested, along with two strains known for their high thermoadaptation success, to ensure that the reduced cycle still allows thermoadaptation. Following the shortened thermoadaptation cycle, strains 01-B526 and SHY16-3432 showed viability at 18 °C following the first subculture, an outcome not previously observed with the 76 h cycle. However, subsequent subcultures revealed that these bacteria were not truly thermoadapted. In contrast, the positive control strains successfully produced thermoadapted strains, confirming that reduced stress can be sufficient to induce thermoadaptation in certain strains while also supporting bacterial survival. Despite initial viability following temperature fluctuations, some strains appear to only undergo temporary adaptation, without the genomic changes required for a stable phenotypic shift.

**Figure 4:**
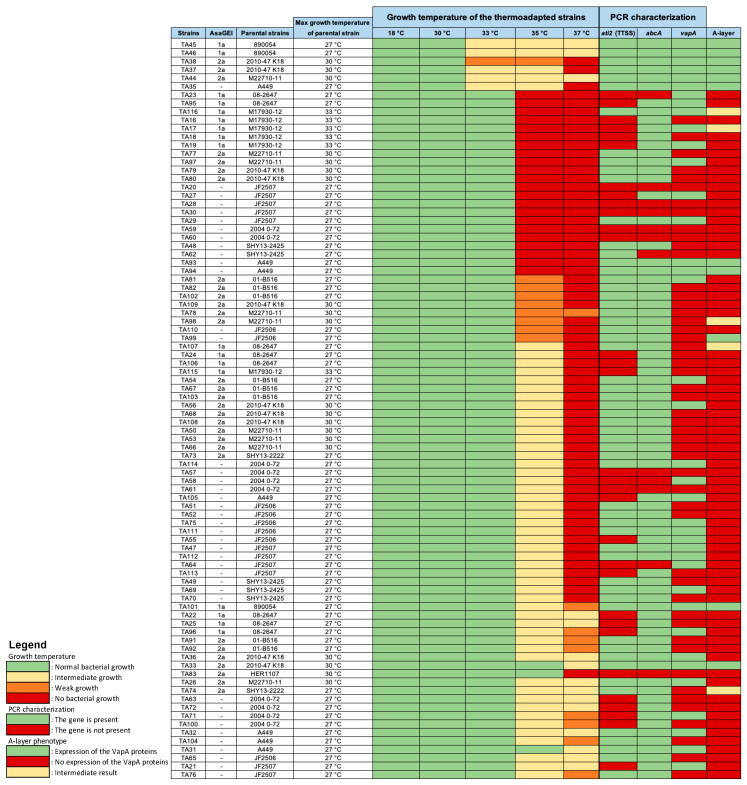
Collection of thermoadapted strains created for this study.

To gain deeper insight into the consequences of the stressful growth conditions that led to thermoadaptation, we analyzed the thermoadapted strains to identify potential shared features or patterns. A series of phenotypic and genotypic analyses were performed on the collection of 88 thermoadapted strains. Growth was assessed on solid medium at five different temperatures (18, 30, 33, 35, and 37 °C) to evaluate thermal tolerance profiles (Figure 4). All strains showed pigmentation only at 18 °C. This result is expected since the production of the characteristic melanin pigment in *A. salmonicida* subsp. *salmonicida* is temperature-dependent. It is produced at low temperatures (≤22 °C) and this production decreases and is inhibited at higher temperatures (30 °C and above) due to the thermolability of the activity of the enzyme HppD (4-hydroxyphenylpyruvate dioxygenase) involved in the melanogenesis process [41,42]. Mutations that increase the thermostability of HppD can restore pigment production at elevated temperatures [3,4]. Here, despite their adaptation to temperatures above 30 °C, the thermoadapted strains still exhibited a loss of pigmentation similar to the parental strains, suggesting that HppD activity remains thermolabile even after thermoadaptation. Among the 88 thermoadapted strains, 64 (72%) exhibited low or intermediate growth up to 35 °C, while 24 (29%) displayed also little growth at 37 °C. If we consider the maximum growth temperature of the parental strains used in this study that varied on solid media from 27 °C for most of the cases to 33 °C in some cases, this gain can represent up to 10 °C in some cases (Figure 4). The difference in growth capacity between thermoadapted and parental strains can also be observed in liquid culture. As an example, Figure S2 shows that the thermoadapted strain TA104 reached an optical density above 0.7 at 595 nm within about 10 h before plateauing, whereas its parental strain A449, from which TA104 was derived, displayed no significant growth under the same conditions.

**Figure 5:**
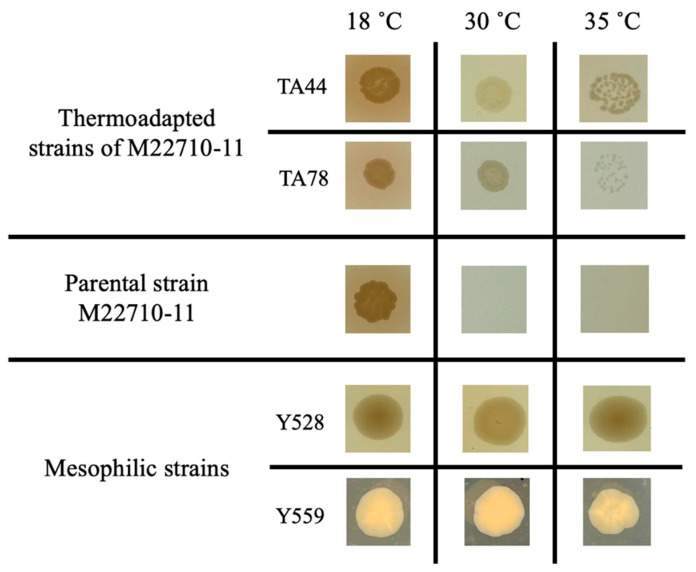
Comparison of the growth of *A. salmonicida* strains on TSA medium at three distinct temperatures, including thermoadapted strains, their parental strain, and mesophilic strains. The cultures were inoculated using a 10^−5^ dilution from an initial bacterial suspension standardized to have an optical density at 600 nm of 1.0.

Growth on the solid medium also revealed that thermoadapted strains did not mimic mesophilic growth patterns. As shown in Figure 5, the biomass produced by the thermoadapted strains is always lower than mesophilic strains. These results confirm that thermoadaptation of these bacteria is not equivalent to them having a mesophilic lifestyle.

**Figure 6:**
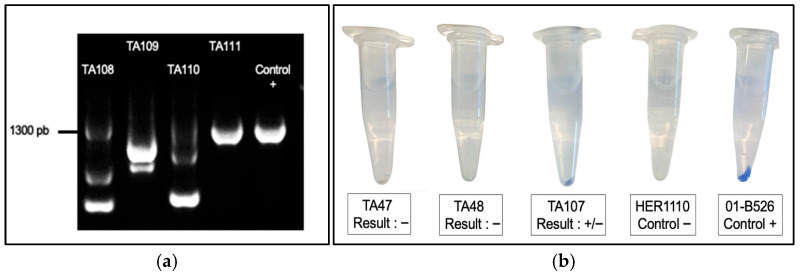
The *vapA* gene and the A-layer phenotype in thermoadapted strains. (**a**) PCR amplification results of the *vapA* gene show variability. (**b**) Phenotypic assay for A-layer detection; blue pellets indicate VapA expression, white pellets indicate absence.

The impact of thermoadaptation on virulence was assessed by examining the presence of the TTSS in thermoadapted strains. This was done using PCR targeting the *ati2* gene, a TTSS marker located on the pAsa5 plasmid [5,23]. Loss of the *ati2* gene indicated a rearrangement of the pAsa5 plasmid and excision of the TTSS [24]. Among all thermoadapted strains, 35% showed loss of this key virulence factor (Figure 4). This result is fully consistent with the findings of Marcoux et al. (2020) [24], who observed pAsa5 rearrangements exclusively in North American strains carrying *AsaGEI1a* and in strains from Europe (Figure 5). In their study, the rearrangement was induced by prolonged growth at 25 °C for six days [24]. The fact that a similar outcome was achieved here using a shorter but more intense thermal stress suggests that the pAsa5 rearrangement is not restricted to a single type of stress condition. We also verified the presence and expression of the A-layer in thermoadapted strains. PCR characterization showed that a majority of thermoadapted strains (50/88) exhibited either mutations or loss of the *vapA* gene, as indicated by amplicon patterns that differed from the positive control, often displaying multiple bands (Figure 6a). There could be, following the heat stress, a partial duplication of the gene encoding the A-layer proteins at another location in the genome, which may be a way for the bacterium to adapt to a particular environment [43]. A phenotypic assay revealed that only 13% of strains expressed a functional A-layer on the membrane surface, with some showing an intermediate phenotype (Figure 6b). Despite the inherent variability linked to the quality of washes [39], proper controls confirmed the reliability of the results. Variability in thermoadapted strains likely reflects differences in VapA abundance covering the surface of the bacterium, which may be explained by defects in VapA transport, *vapA* expression, or LPS structure affecting A-layer anchoring.

The A-layer is known to be thermolabile in *A. salmonicida* subsp. *salmonicida* [9,25], although the underlying mechanisms remain poorly understood. Several hypotheses have been proposed: the A-layer may affect outer membrane fluidity, and its loss could help maintain membrane integrity at elevated temperatures. Additionally, the processes of A-layer biosynthesis and protein export have a significant metabolic cost, which may be unfavorable under thermal stress [44,45]. In this study, genes associated with A-layer formation (*vapA* and *abcA*) were found to be prone to mutations during thermoadaptation. Even though 13% of the strains display a functional A-layer, these findings suggest a potential link between A-layer loss and thermoadaptation, but the specific genetic or regulatory mechanisms driving this loss remain to be elucidated.

Thermoadaptation assays revealed that this process appears to be largely stochastic in *A. salmonicida* subsp. *salmonicida*, even among parental strains that show thermoadaptive potential (Figure 3). The variability observed between replicates is likely attributable to spontaneous mutations arising within bacterial populations, introducing genetic changes over time. While elevated temperatures create stressful conditions that can compromise bacterial survival [46], they may also accelerate evolutionary processes by increasing the mutation rate, which typically ranges from 1 × 10^−5^ to 1 × 10^−8^ per cell division [47,48]. As a result, some bacteria may successfully acclimate through the random acquisition of beneficial mutations—though the inherent randomness of these events introduces an element of unpredictability to the adaptation process. The random nature of thermoadaptation is further supported by the observation that the same parental strain can give rise to thermoadapted derivatives with widely varying characteristics. For instance, the upper growth temperature limits among thermoadapted strains can range from 33 °C to 37 °C, with or without concurrent loss of key virulence factors. Strain A449 provides a striking example of this phenomenon, having generated a highly diverse set of thermoadapted variants (Figure 4). These findings underscore the complexity of thermoadaptation and suggest that multiple mutational pathways may lead to similar outcomes.

A detailed molecular understanding of this process will require sequencing a large number, potentially hundreds, of thermoadapted genomes to identify recurrent genetic changes involved in adaptation. Generating extensive thermoadapted derivative libraries from a single parental strain such as A449 could serve as a strategic approach, though it is important to recognize that such efforts would only begin to reveal the full extent of the underlying complexity.

Additionally, our assays revealed that some strains appear entirely incapable of adapting to elevated temperatures. This outcome was not unexpected, given that the thermal variation cycle used in this study does not reflect gradual environmental shifts. While aquatic temperatures do fluctuate, especially on a seasonal basis, bacteria in nature are typically not subjected to abrupt temperature increases over short timeframes. In contrast, the rapid shifts applied in this experimental design require immediate acclimation, a challenge that some strains may be genetically unequipped to meet. This suggests that certain genetic elements or regulatory systems may actively constrain the capacity for rapid thermoadaptation.

Finally, although thermoadaptation of *A. salmonicida* subsp. *salmonicida* might appear to have implications for aquaculture, its direct relevance in this context is limited. Indeed, the fish species naturally infected by this pathogen do not tolerate the elevated temperatures considered in this study, and we observed that thermoadapted strains often lost major virulence factors, further reducing their pathogenic potential. Nevertheless, the ability of some strains to permanently adapt to higher temperatures raises the possibility that *A. salmonicida* subsp. *salmonicida* could persist in warmer aquatic environments, which are expected to become more common under climate change scenarios. Such ecological consequences remain speculative at this stage, but they highlight the need to further explore how thermal stress may influence the long-term dynamics of bacterial populations in natural waters. In addition, the altered virulence profiles observed in several thermoadapted strains suggest that they could represent promising candidates for future exploration as live attenuated vaccines against furunculosis, the disease caused by *A. salmonicida* subsp. *salmonicida*.

## 4. Conclusions

In this study, we demonstrated that the upper temperature growth limit of *A. salmonicida* subsp. *salmonicida* can be permanently shifted through thermoadaptation. Notably, this adaptive process appears to be strain-dependent: strains carrying the genomic island *AsaGEI1a* were more resistant to thermoadaptation. However, the underlying genomic mechanisms driving this adaptation are likely complex, as suggested by the diverse phenotypic and genotypic profiles observed among the thermoadapted strains generated during the trials. To gain a more comprehensive understanding of these processes, it will be necessary to sequence a substantial number of genomes from both parental and thermoadapted strains.

Finally, our findings corroborate those of previous reports, which suggest that the A-layer and the TTSS are lost during a thermal stress. At present, it remains unclear whether this loss is an adaptive mechanism that supports thermoadaptation or instead an incidental consequence of the genomic plasticity of this bacterium. Future work should aim to unravel these possibilities, while also assessing how thermoadapted strains could be exploited for aquaculture applications for the development of live attenuated vaccines.

## Figures and Tables

**Table 1:** Genes exclusive to the group of strains that harbor *AsaGEI1a*.

Gene Product	Gene Position in *A. salmonicida* subps. *salmonicida* Strain A449 (Reference)
Glycosyltransferase family 2 protein	166543-166713
*TniA* transposase (partial)	1073012-1073191

## Data Availability

The datasets generated and analyzed during the current study are available in the GenBank repository as described in Table S2. The sequence of *AsaGEI1a* from strains M13566-12 has been deposited in the Genbank (accession number PV737463). The chromosomal sequence of strain M17930-12 has been deposited on Genbank (accession number PRJNA1269977).

## Supplementary Materials

The following supporting information can be downloaded at: https://www.mdpi.com/article/10.3390/microorganisms13092171/s1. Figure S1: Shorter version (38 h) of the thermoadaptation cycle; Table S1: Strains of *Aeromonas salmonicida* subps. *salmonicida* used for this study; Table S2: Strains of *Aeromonas salmonicida* subps. *salmonicida* used for the genomic analysis; Table S3: Primers used in this study [49,50,51,52,53,54,55,56,57].
