# Supplementary material for: Strain-Dependent Thermoadaptation in the Fish Pathogen Aeromonas salmonicida subsp. salmonicida
Source: Microorganisms. 2025 Sep 17;13(9):2171. doi: 10.3390/microorganisms13092171 (PMC12472413; doi:10.3390/microorganisms13092171)
Supplement: Supplementary file 1 [file microorganisms-13-02171-s001.zip › microorganisms-3820220-supplementary.pdf]

SUPPLEMENTARY DATA

Strain-dependent thermoadaptation in the fish pathogen *Aeromonas salmonicida* subsp. *salmonicida*

Kim C. Fournier[1,2], Pierre-Étienne Marcoux[1,2], Antony T. Vincent[1,3] et Steve J. Charette[1,2,*]

1. Institut de Biologie Intégrative et des Systèmes, Université Laval, Quebec City, QC, G1V 0A6, Canada.
2. Département de Bicohimie, de Microbiologie et de Bio-Informatique, Faculté des Sciences et de Génie, Université Laval, Quebec city, Qc, G1V 0A6, Canada.
3. Département des Sciences Animales, Faculté des Sciences de l'Agriculture et de l'Alimentation, Université Laval, Quebec City, QC, G1V 0A6, Canada.
*Correspondence: steve.charette@bcm.ulaval.ca

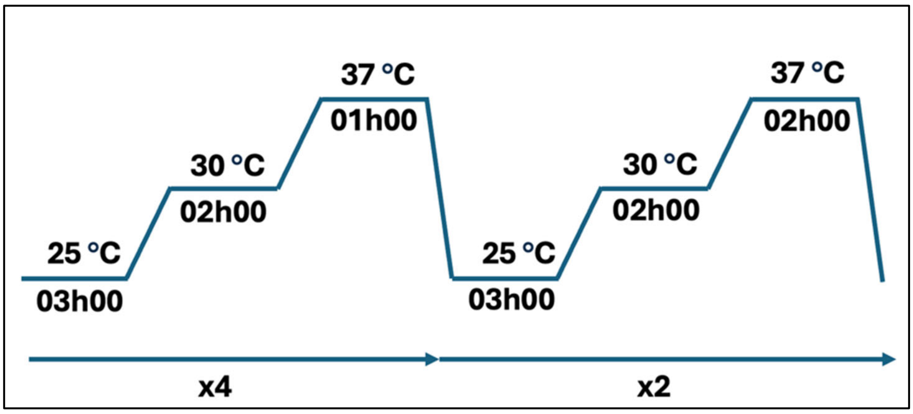

Figure S1. Shorter version (38 hours) of the thermoadaptation cycle.

Table S1. Strains of *Aeromonas salmonicida* subps. *salmonicida* used for this study.

Type of *AsaGEI*	Strain	Presence of the A-layer	Prophage 3	Origin	Reference
**Psychrophilic strain with *AsaGEI1a***	01-B526	+	-	Quebec, Canada	[1]
	08-2647	+	-	Quebec, Canada	[2]
	M9954-10	+	-	Quebec, Canada	[3]
	M15879-11	+	-	Quebec, Canada	[3]
	M13566-12	+	-	Quebec, Canada	[4]
	M17930-12	+	-	Quebec, Canada	[4]
	SHY15-4029	+	-	Quebec, Canada	[5]
	SHY16-3012	+	-	Quebec, Canada	[6]
	SHY16-3432	+	-	Quebec, Canada	[7]
	SHY18-3337	+	-	Quebec, Canada	[8]
	SHY20-1590	+	-	Quebec, Canada	[6]
	SHY22-5948	+	-	Quebec, Canada	[9]
	890054	+	-	USA	[10]
**Psychrophilic strain with *AsaGEI2a***	01-B516	+	+	Quebec, Canada	[2]
	M22710-11	+	+	Quebec, Canada	[3]
	SHY13-2222	+	+	Quebec, Canada	[4]
	2010-47 K18	+	+	New Brunswick, Canada	[11]
**Psychrophilic strain with No *AsaGEI***	A449	+	-	France	[12]
	JF2506	+	-	Norway	[4]
	JF2507	+	-	Norway	[4]
	2004-072	+	-	British Columbia, Canada	[13]
	SHY13-2425	+	-	Quebec, Canada	[14]
**Mesophilic strain with No *AsaGEI***	Y528	N/A	N/A	India	[15]
	Y559	N/A	N/A	India	[15]

Table S2. Strains of *Aeromonas salmonicida* subps. *salmonicida* used for the genomic analysis.

Type of *AsaGEI*	Strain	Origin	Reference	Accession number
AsaGEI1a	01-B526	Quebec, Canada	[1]	GCA_003692675.1
	M15879-11	Quebec, Canada	[3]	GCA_002110585.1
	SHY16-3432	Quebec, Canada	[7]	GCA_008370735.1
	890054	USA	[10]	GCA_020683025.1
AsaGEI2a	M22710-11	Quebec, Canada	[3]	GCA_002811185.1
	2004-05MF26	New Brunswick, Canada	[11]	GCA_000786805.1
	M16474-11	Quebec, Canada	[3]	GCA_005476635.1
	2009-157 K5	New Brunswick, Canada	[11]	GCA_001902045.1
	2010-47 K18	New Brunswick, Canada	[11]	GCA_001902055.1
**No *AsaGEI***	A449	France	[12]	GCA_000196395.1
	RS534	France	[16]	GCA_001499805.2
	JF3517	Switzerland	[4]	GCA_001902125.1
	JF2506	Norway	[4]	GCA_001902065.1
	JF2507	Norway	[4]	GCA_001902105.1
	2004-072	British Columbia, Canada	[17]	GCA_016811055.1

Table S3. Primers used in this study.

Target	Sequence (5'-3') Fw/Rev	PCR product (bp)
vapA	CAGGACATGAGCATCAGTAGTTTCC / CGACTAGATTCGCTCTTACAGAGTG	1669
abcA	GTTCCCTCACTCATCAATCTCTT / ACTCAGTCTGGCTGGTATCT	1724
ati2	ACACGATGATACGCACCTAGCCAA / TTGACCTGTGGTCAGGTTAGCAGT	550

REFERENCES

1. Dautremepuits, C.; Fortier, M.; Croisetiere, S.; Belhumeur, P.; Fournier, M. Modulation of juvenile brook trout (*Salvelinus fontinalis*) cellular immune system after *Aeromonas salmonicida* challenge. *Vet. Immunol. Immunopathol.* **2006**, *109*, 65–74. DOI: 10.1016/j.vetimm.2005.09.008.
2. Daher, R.K.; Filion, G.; Tan, S.G.; Dallaire-Dufresne, S.; Paquet, V.E.; Charette, S.J. Alteration of virulence factors and rearrangement of pAsa5 plasmid caused by the growth of *Aeromonas. salmonicida.* in stressful conditions. *Vet Microbiol* **2011**, *152*, 353–360. https://doi.org/10.1016/j.vetmic.2011.04.034.
3. Trudel, M.V.; Tanaka, K.H.; Filion, G.; Daher, R.K.; Frenette, M.; Charette, S.J. Insertion sequence AS5 (ISAS5) is involved in the genomic plasticity of *Aeromonas salmonicida*. *Mob. Genet. Elements* **2013**, *3*, e25640.
4. Attéré, S. A.; Vincent, A. T.; Trudel, M. V.; Chanut, R.; Charette, S. J. Diversity and homogeneity among small plasmids of *Aeromonas salmonicida* subsp. *salmonicida* linked with geographical origin. *Front Microbiol* **2015**, *6*, 1274. DOI: 10.3389/fmicb.2015.01274.
5. Attéré, S.A.; Vincent, A.T.; Paccaud, M.; Frenette, M.; Charette, S.J. The role for the small cryptic plasmids as moldable vectors for genetic innovation in *Aeromonas salmonicida* subsp. *salmonicida*. *Front. Genet.* **2017**, *8*, 211. https://doi.org/10.3389/fgene.2017.00211.
6. Marcoux, P.; Girard, S.B.; Fournier, K.C.; Tardif, C.A.; Gosselin, A.; Charette, S.J. Interaction of pAsa5 and pAsa8 Plasmids in *Aeromonas salmonicida* subsp. *salmonicida*. *Microorganisms* **2023**, *11*, 2685. https://doi.org/10.3390/microorganisms11112685.
7. Massicotte, M.-A.; Vincent, A.T.; Schneider, A.; Paquet, V.E.; Frenette, M.; Charette, S.J. One *Aeromonas salmonicida*subsp. *salmonicida* isolate with a pAsa5 variant bearing antibiotic resistance and a pRAS3 variant making a link with a swine pathogen. *Microbiol. Resour. Announc.* **2022**, *11*, e01092-21. DOI: 10.1128/mra.01092-21.
8. Marcoux, P.; Vincent, A.T.; Massicotte, M.A.; Paquet, V.E.; Doucet, É., J.; Hosseini, N.; Trudel, M.V.; Byatt, G.; Laurent, M.; Frenette, M.; et al. Systematic analysis of the stress-induced genomic instability of type three secretion system in *Aeromonas salmonicida* subsp. *salmonicida*. *Microorganisms* **2020**, *9*, 85. https://doi.org/10.3390/microorganisms9010085.
9. Girard, S.B.; Marcoux, P.-É.; Paquet, V.E.; Zoubaï, S.; Can, T.N.V.; Attéré, S.A.; Vincent, A.T.; Charette, S.J. Expansion of the tetracycline resistome in *Aeromonas salmonicida* with a tet(D) gene found in plasmids pAsa-2900 and pAsa-2900b. *Front. Bacteriol.* **2024**, *3*, 1418706. DOI: 10.3389/fbrio.2024.1418706.
10. Gauthier, J.; Marquis, H; Paquet, V.E.; Charette, S.J.; Levesque, R.C.; Derome, N. Genomic perspectives on *Aeromonas salmonicida* subsp. *salmonicida* strain 890054 as a model system for pathogenicity studies and mitigation of fish infections. *Front. Mar. Sci.* **2021**, *8*, 744052. DOI: 10.3389/fmars.2021.744052.
11. Vincent, A.T.; Trudel, M.V.; Paquet, V.E.; Boyle, B.; Tanaka, K.H.; Dallaire-Dufresne, S.; Daher, R.K.; Frenette, M.; Derome, N.; Charette, S.J. Detection of variants of the pRAS3, pAB5S9, and pSN254 plasmids in *Aeromonas salmonicida* subsp. *salmonicida*: Multidrug resistance, interspecies exchanges, and plasmid reshaping. *Antimicrob. Agents Chemother.* **2014**, *58*, 6879–6886.
12. Dacanay, A.; Knickle, L.; Solanky, K.S.; Boyd, J.M.; Walter, J.A.; Brown, L.L.; Johnson, S.C.; Reith, M. Contribution of the type III secretion system (TTSS) to virulence of *Aeromonas salmonicida* subsp. *salmonicida*. *Microbiology* **2006**, *152 Pt 6*, 1847–1856. https://doi.org/10.1099/mic.0.28768-0.
13. Vaillancourt, K.C.; Paquet, V.E.; Vincent, A.T.; Schneider, A.; Thompson, C.; Laurent, M.; Frenette, M.; Charette, S.J. Draft genome sequence of an *Aeromonas salmonicida* subsp. *salmonicida* strain from the Canadian Pacific Coast bearing a variant of pRAS1. *Microbiol. Resour. Announc.* **2021**, *10*, DOI: 10.1128/MRA.00291-21.

14. Trudel, M. V.; Vincent, A. T.; Attéré, S. A.; Labbé, M.; Derome, N.; Culley, A. I.; Charette, S. J. Diversity of antibiotic-resistance genes in Canadian isolates of *Aeromonas salmonicida* subsp. *salmonicida*: dominance of pSN254b and discovery of pAsa8. *Scientific reports* **2016**, *6*, 35617-35617. DOI: 10.1038/srep35617.
15. Vanden Bergh, P.; Frey, J. *Aeromonas salmonicida* subsp. *salmonicida* in the light of its type-three secretion system. *Microb. Biotechnol.* **2014**, *7*, 381–400. https://doi.org/10.1111/1751-7915.12091.
16. Vincent, A.T.; Trudel, M.V.; Freschi, L.; Nagar, V.; Gagné-Thivierge, C.; Levesque, R.C.; Charette, S.J. Increasing genomic diversity and evidence of constrained lifestyle evolution due to insertion sequences in *Aeromonas salmonicida*. *BMC Genom.* **2016**, *17*, 44. https://doi.org/10.1186/s12864-016-2381-3.
17. Vaillancourt, K.C.; Paquet, V.E.; Vincent, A.T.; Schneider, A.; Thompson, C.; Laurent, M.; Frenette, M.; Charette, S.J. Draft genome sequence of an *Aeromonas salmonicida* subsp. *salmonicida* strain from the Canadian Pacific Coast bearing a variant of pRAS1. *Microbiol. Resour. Announc.* **2021**, *10*, DOI: 10.1128/MRA.00291-21.
